# Cholesterol Modulates the Interaction between HIV-1 Viral Protein R and Membrane

**DOI:** 10.3390/membranes11100784

**Published:** 2021-10-13

**Authors:** Chun-Hao Liu, Shing-Jong Huang, Tsyr-Yan Yu

**Affiliations:** 1 Chemical Biology and Molecular Biophysics, Taiwan International Graduate Program, Academia Sinica, Taipei 11529, Taiwan; gerard1057@gmail.com; 2 Institute of Bioinformatics and Structural Biology, National Tsing Hua University, Hsin Chu 300044, Taiwan; 3 Institute of Atomic and Molecular Sciences, Academia Sinica, Taipei 10617, Taiwan; 4 Instrumentation Center, National Taiwan University, Taipei 10617, Taiwan; 5 International Graduate Program of Molecular Science and Technology, National Taiwan University, Taipei 10617, Taiwan

**Keywords:** HIV-1, Vpr, cholesterol, lipid composition, membrane, calcein release, REDOR, NMR

## Abstract

Being a major metabolite for maintaining cellular homeostasis, as well as an important structural component in lipid membrane, cholesterol also plays critical roles in the life cycles of some viruses, including human immunodeficiency virus-1 (HIV-1). The involvement of cholesterol in HIV-1 infectivity, assembly and budding has made it an important research target. Viral protein R (Vpr) is an accessory protein of HIV-1, which is involved in many major events in the life cycle of HIV-1. In addition to its multi-functional roles in the HIV-1 life cycle, it is shown to interact with lipid membrane and form a cation-selective channel. In this work, we examined the effect of cholesterol on the interaction of Vpr and lipid membrane. Using calcein release assay, we found that the membrane permeability induced by the membrane binding of Vpr was significantly reduced in the presence of cholesterol in membrane. In addition, using solid-state NMR (ssNMR) spectroscopy, Vpr was shown to experience multiple chemical environments in lipid membrane, as indicated by the broad line shape of carbonyl ^13^C resonance of Cys-76 residue ranging from 165–178 ppm, which can be attributed to the existence of complex Vpr-membrane environments. We further showed that the presence of cholesterol in membrane will alter the distribution of Vpr in the complex membrane environments, which may explain the change of the Vpr induced membrane permeability in the presence of cholesterol.

## 1. Introduction

Cholesterol is generally viewed as a structural component in cell membranes, which plays an important role in membrane stability and fluidity. It is also an essential precursor for the biosynthesis of several steroid hormones and bile acids [1]. Recently, the roles of cholesterol in the life cycles of various viruses, in the aspects of viral infectivity, assembly, and structural stability, have attracted significant attention [2]. High cholesterol content of influenza virus particles was reported to be a critical factor in the viral fusion process [3]. Lipid rafts, containing high cholesterol levels, were shown to be essential in the assembly of viral particles of parainfluenza virus type 3 [4]. For HIV-1, the viral particle contains high levels of cholesterol [5,6] and the involvement of cholesterol in HIV-1 infectivity, assembly, budding, etc., has been reported [7,8,9,10,11,12,13].

HIV-1 viral protein R (Vpr) is a functional accessory protein, which is involved in many aspects of the viral life cycle, including the nuclear import of the pre-integration complex [14,15,16], cell cycle arrest at G2 phase [17], cell apoptosis [18,19,20], transactivation of the long-terminal repeat (LTR) genes [21], etc. [22,23,24,25]. In addition to its functional roles in HIV-1 viral cycle, the interactions between Vpr and lipid membrane also draw significant attention. Vpr could form a cation-selective channel on lipid membrane [23]. The peptides derived from Vpr sequence were demonstrated to behave as cell-penetrating peptides (CPPs), showing the potential applications such as DNA transfection and gene delivery [26,27,28]. Previously, we reported a novel strategy for producing Vpr protein in large quantity using an *E. coli* expression system and further demonstrated that Vpr has a stronger binding affinity to DOPG lipids compared to DOPC lipids, suggesting a lipid-composition dependent of Vpr-membrane interaction [29]. During the HIV-1 virus particle assembly at the plasma membrane, lipid rafts containing high amount of cholesterol are targeted by Gag proteins [9,10]. In addition, depletion of cellular cholesterol significantly reduces HIV-1 particle production [30]. Once the viral particles are released out from the lipid raft, a higher cholesterol content in the HIV-1 virus envelope may benefit to its stability [31]. Moreover, significant amount of Vpr proteins are packaged into the virus particle by Vpr/Gag ratio to be around 1:7 [32], which contribute to the early infection of the virus. Its association with the p6 protein is essential for the translocation from the nuclear membrane to the plasma membrane as well as packaging into the virus particle [33,34]. Thus, it is reasonable to investigate if cholesterol, being an important structural component in the plasma membrane, may play a role in the interaction of Vpr and membrane. We first used calcein release assay to probe the effect of cholesterol on Vpr induced membrane permeability and showed that the Vpr induced membrane permeability was reduced as the content of cholesterol in membrane was increased. This result is similar to what were observed from the studies of a pore-forming peptide [35] and CPPs [36]. The analysis using circular dichroism (CD) spectroscopy showed that no significant change of the secondary structure of Vpr was observed with the addition of cholesterol in Vpr proteoliposomes. NMR spectroscopy is a straightforward tool for acquiring residue specific information of the binding event. In addition, the chemical shift of a NMR resonance signal is a result of local electron shielding, and thus, it reflects the local chemical environment. Solution NMR was used here to examine if Vpr protein has specific interaction with cholesterol. As a result, we did not observe specific binding of cholesterol to Vpr in DPC micelles. Even though cholesterol does not bind to Vpr specifically, nor affect the secondary structure directly, cholesterol may still modulate the Vpr-membrane interaction by changing the protein-lipid complex arrangement. REDOR NMR is a powerful solid-state NMR method for providing the distance information between two hetero nuclei. Through our ssNMR study of [1-^13^C, 99%] Cysteine selective labeled Vpr embedded in liposome, we showed the existence of complex Vpr-membrane environments and the distribution of Vpr among these environments was altered with the presence of cholesterol in membrane. We believe that more insights into cholesterol modulating Vpr-membrane interaction can be revealed by other methods, such as computational simulations. In summary, we show that cholesterol, without interacting with Vpr directly, can affect the Vpr-membrane interaction.

## 2. Materials and Methods

### 2.1. Materials

1,2-dioleoyl-sn-glycero-3-phospho-(1′-rac-glycerol) (DOPG) and 1,2-dioleoyl-sn-glycero-3-phosphocholine (DOPC) were purchased from Avanti Polar Lipids (Alabaster, AL, USA). [^15^N, 99%] ammonium chloride, [U-^13^C, 99%] D-glucose, and [1,1′-^13^C_2_, 99%] L-Cystine were purchased from Cambridge Isotope Laboratories (Andover, MA, USA). [^2^H_25_, 98%] dodecylphosphocholine (DPC) was purchased from Frankenstein Bio Reagents, (Cambridge, MA, USA). Chloroform was purchased from Avantor, Radnor, PA, USA. 2,2,2-Trifluoroethanol (TFE) was purchased from Alfa Aesar, Hersham, UK. 2,2,2-Trifluoroacetic acids (TFA) was purchased from ACROS Organics, Morris Plains, NJ, USA. Deuterium oxide (D_2_O), Triton X-100 (TX-100), calcein, cholesterol, 3-(trimethylsilyl)-1-propanesulfonic acid sodium salt (DSS) were purchased from Sigma Aldrich, St. Louis, MO, USA. ISOGRO was purchased from Silantes, Munich, DE or Sigma Aldrich, St. Louis, MO, USA.

### 2.2. Vpr Protein Production and Purification

We followed the established protocol [29] to produce, purify and characterize HIV-1 Vpr protein. Briefly, an overnight culture of BL21(DE3) *E. coli* (RBC Bioscience, Birmingham, UK), transformed with His-tagged B1 domain of streptococcal protein G (GB1)-fused Vpr construct, was prepared at 37 °C in LB medium, and then sub-cultured at 20 °C in a defined growth medium described in the supporting information of reference number 29. To prepare [U-^2^H,^13^C,^15^N] labeled Vpr protein, 1 g of [^15^N, 99%] ammonium chloride, 2 g of [U-^13^C, 99%] D-glucose and 1 L of D_2_O (98%) per liter culture were used to replace the natural abundance chemicals in the growth medium. This culture was also supplemented with the 1 g of [U-^13^C, ^15^N] ISOGRO per liter after induction with 0.1 mM isopropyl β-D-thiogalactoside (IPTG). To prepare [1-^13^C, 99%] Cysteine selective labeled Vpr protein, a mixture of amino acids and nucleobases was used to replace the ammonium chloride in the defined growth medium. The mixture of amino acids and nucleobases contains 125 mg of [1,1′-^13^C_2_, 99%] L-Cystine, 200 mg of each of the rest of nineteen non-labeled amino acids, and the five nucleobases, including 125 mg of adenine, 125 mg of guanine, 125 mg of thymine, 125 mg of cytosine, and 50 mg thymine per liter.

The culture was induced with 0.1 mM IPTG, while the OD_600_ reached the range of 0.7–0.9, and the culture was continued to grow at 18 °C for 24 h to allow for the expression of the soluble His-tagged GB1-fused Vpr protein. Pure Vpr protein precipitates can be obtained following the established protocol [29]. The Vpr protein was examined by sodium dodecyl sulfate polyacrylamide gel electrophoresis (SDS-PAGE) and matrix-assisted laser desorption ionization-time of flight (MALDI-TOF) mass spectra (microflex LRF, Bruker, MA, USA) to ensure the purity as shown in an example in Figure S1.

### 2.3. DPC Solubilized Vpr Sample for Sequence-Specific Resonance Assignments

The Vpr protein precipitates were obtained by treating [U-^2^H,^13^C,^15^N] labeled His-tagged GB1-fused Vpr with tobacco etch virus (TEV) protease to remove His-tagged GB1 tag. The [U-^2^H,^13^C,^15^N] labeled Vpr protein precipitates dissolved in a denaturing buffer, consisting of 50 mM sodium phosphate at pH 6.5, 50 mM sodium chloride (NaCl), 0.5 mM tris(2-carboxyethyl)phosphine (TCEP), 1 mM ethylenediaminetetraacetic acid (EDTA), and 6 M guanidine hydrochloride (GdnHCl), to the concentration of 1 mg/mL. The protein solution was then added in a dropwise manner at a flow rate of 50 µL/min to a refolding buffer, consisting of 50 mM sodium phosphate at pH 6.5, 50 mM NaCl, 0.5 mM TCEP, 1 mM EDTA, and 9 mM of the tail deuterated [^2^H_25_, 98%] DPC. The protein solution was further dialyzed against a buffer containing 25 mM sodium phosphate at pH 6.5, 25 mM NaCl, 1 mM dithiothreitol (DTT), and 1 mM EDTA at 4 °C using a 6–8 kDa molecular weight cut-off (MWCO) dialysis membrane (Orange Scientific, Braine-l’Alleud, Belgium). The protein solution was concentrated and further purified by size exclusion chromatography using a Superdex increase s200 (10/300) GL column (Cytiva, Marlborough, MA, USA) equilibrated with a buffer containing 25 mM sodium phosphate at pH 6.5, 25 mM NaCl, 1 mM TCEP, 1 mM EDTA, and 1.8 mM [^2^H_25_, 98%] DPC. The NMR samples were further concentrated with 5 kDa molecular weight cut-off (MWCO) centricon (Vivaspin, Göttingen, Germany). The samples for the 3D triple resonance experiments and for the cholesterol titration experiment were of the concentrations of 552 µM and 280 µM, respectively. D_2_O, containing DSS at the concentration of 20 mg/mL, was included in each NMR sample to 6% (wt %).

### 2.4. Vpr Proteoliposomes Preparation

To produce Vpr incorporated in DOPG liposomes, DOPG lipid was dissolved in chloroform (50 mg/mL) and dried on the side of a round bottom flask using a gentle stream of nitrogen gas. To produce Vpr incorporated in DOPG containing 5%, 10%, and 30% of cholesterol liposomes, the lipid mixes were first solubilized in chloroform with the molar ratio of DOPG lipid to cholesterol as 95 to 5, 90 to 10 and 70 to 30, respectively. Vpr protein precipitates were dissolved in a TFE solution containing 0.1% TFA to the concentration of 1 mg/mL, and then added to the dried lipid film containing flask. The lipid and protein mixture were prepared with the molar ratio of lipid to Vpr as 50 to 1. A gentle stream of nitrogen gas was used to remove the organic solvent, followed by placing the flask under vacuum overnight. The protein-lipid film formed on the glass wall was re-suspended in a buffer containing 5 mM sodium phosphate at pH 7.5, 5 mM potassium sulfate, and 1 mM TCEP. The Vpr proteoliposomes were further homogenized using an extruder with polycarbonate membrane with a defined pore size of 0.4 µm in diameter. For secondary structure analyses using circular dichroism spectroscopy, the Vpr proteoliposomes were purified by size exclusion chromatography using an ENrich 70 (10/300) column (Bio-Rad, Hercules, CA, USA) in a buffer containing 5 mM sodium phosphate at pH 7.5, 5 mM potassium sulfate, and 1 mM TCEP. To prepare the samples for the ssNMR experiment, [1-^13^C] cysteine selective labeled Vpr protein was used in the proteoliposomes production, and a DOPG liposome without Vpr protein incorporated was also prepared as a reference ssNMR sample. The size distribution of Vpr proteoliposomes were characterized by dynamic light scattering to ensure the size homogeneity (DelsaNano C particle size and zeta potential analyzer, Beckman Coulter, Indianapolis, IN, USA), as indicated in Figure S2A–F.

### 2.5. Calcein Release Assay

We used calcein release assay to study the Vpr induced membrane permeabilization of liposomes [29]. When the membrane-impermeable fluorescent probe calcein is encapsulated in liposome, the fluorescence signal is low due to the self-quenching effect of calcein at high concentration. The fluorescence signal will increase accompanied by the release of calcein from liposomes. To examine the effect of cholesterol on the Vpr induced membrane permeabilization, we prepared liposomes containing various amount of cholesterol. The four different liposomes were prepared with the four different molar ratios of cholesterol to lipid, including 20 to 80, 30 to 70, 40 to 60 and 50 to 50, while an equal amount of DOPC lipid and DOPG lipid were used in each liposome preparation. The liposomes were prepared in a buffer containing 25 mM Tris at pH 7.5, 25 mM NaCl, 1 mM TCEP, and 0.5 mM EDTA. The size homogeneity of calcein encapsulated liposomes with increasing cholesterol content were confirmed using dynamic light scattering as shown in Figure S2G–J.

Time-resolved fluorescence spectra were recorded using a luminescence spectrometer (LS55, PerkinElmer, Waltham, MA, USA), to monitor the increase of the fluorescence signal due to the release of calcein from liposomes. The fluorescence spectra at each time point after the addition of His-tagged GB1-fused Vpr to the liposomes solution were recorded from 505 to 535 nm with the excitation light at the wavelength of 495 nm. The interval between each scan was 30 s and the total monitoring time for each experiment was 40 min. The positive control experiments were carried out by recording the time-resolved fluorescence spectra for 40 min after mixing the liposomes solution with a buffer containing 25 mM Tris at pH 7.5, 25 mM NaCl, 1 mM TCEP, 0.5 mM EDTA, and 0.3% TX-100, where the negative control experiments were carried out by monitoring the time-resolved fluorescence spectra of liposomes solution without the addition of His-tagged GB1-fused Vpr or the buffer containing TX-100. Each experimental condition was performed with three replicates.

The calcein release kinetics, quantified by the fluorescence intensity at 517 nm, were quantified using Equation (1).

(1) $$calcein\;release\left(t\right)\%=\frac{I_{Vpr}\left(t\right)\;-\;I_{buffer}\left(t\right)}{I_{TX-100}\left(t\right)\;-\;I_{buffer}\left(t\right)}\times 100\%$$

where *I_Vpr_(t)* is the fluorescence intensity at the time *t* after the addition of His-tagged GB1-fused Vpr to the liposome solution, *I_buffer_(t)* the reference fluorescence intensity at the time *t*, and *I_TX-100_(t)* the fluorescence intensity at the time *t* after the addition of TX-100 containing buffer.

### 2.6. Spectroscopic Characterizations

The CD spectra between 190–260 nm were recorded using Jasco CD spectrometer (J-815, Tokyo, Japan) with a cuvette of 1 mm path length. For each CD spectrum, 8 scans were accumulated and averaged, with the bandwidth of 2 nm, the scanning speed at 50 nm/min and the step resolution of 0.2 nm. The secondary structure content was analyzed by DichroWeb using CDSSTR program and SP175 as reference data set, which was optimized for the range of 190–240 nm. The lowest data point used in the analysis was set to 190 nm, and the scaling factor was 0.95.

For sequence-specific resonance assignment, a set of standard NMR spectra, including 2D [^1^H,^15^N] TROSY-HSQC and 3D TROSY-HNCA, 3D TROSY-HNCOCA, 3D TROSY-HNCACB spectra of [U-^2^H,^13^C,^15^N] Vpr solubilized in [^2^H_25_, 98%] DPC detergent micelles were recorded at 37 °C using Bruker AVANCE III 850 MHz NMR spectrometer (Bruker BioSpin, Billerica, MA, USA) equipped with a TCI (^1^H/^13^C/^15^N) 5mm CryoProbe with Z gradient. The assignment was performed with computer aided resonance assignment (CARA) program [37]. A Vpr sample with the addition of cholesterol at the ratio of cholesterol to Vpr as 1 to 2.6 was prepared and then a 2D [^1^H,^15^N] TROSY-HSQC spectrum was recorded to examine if cholesterol binds specifically to Vpr. To further increase the amount of cholesterol in the sample, additional cholesterol was first dissolved in ethanol to the concentration of 6 mg/mL due to the low solubility of cholesterol in DPC buffer. Then, 10 μL of the cholesterol in ethanol was added to the Vpr sample reaching the final ratio of cholesterol to Vpr as 2.4 to 1. An additional 2D [^1^H,^15^N] TROSY-HSQC spectrum was recorded for the comparison with cholesterol-free spectrum.

To prepare the samples for ssNMR studies, proteoliposome pellets were first collected by ultracentrifugation (Optima XE-90, Beckman Coulter, Indianapolis, IN, USA) at 200,000 g for 4 h. Each pellet was then transferred into a 3.2 mm rotor with the aid of a homemade centrifuge adapter using ultracentrifugation at 200,000 g for 4 h.

All ssNMR experiments were carried out on a Bruker wide-bore 11.7-T Avance III 500 MHz spectrometer (Bruker BioSpin, Billerica, MA, USA) equipped with a 3.2 mm triple-resonance magic angle spinning (MAS) probehead. The samples were cooled down with a nitrogen gas stream under heat exchanging in a 25 L liquid nitrogen dewar and the sample temperature was ca. 233 K. The sample spinning rate was 8 kHz. The ^13^C {^31^P} rotational-echo double-resonance (REDOR) sequence comprises cross polarization (CP) of the ^13^C spin from proton, followed by a series of ^13^C π pulses applied at the end of each rotor cycle for rotational echoes, and a series of ^31^P π pulses at the middle of each rotor cycle for dipolar dephasing. XY-8 phase cycling was applied to both π-pulse chains. The rf field strengths for ^13^C and ^31^P π pulse was 35 and 50 kHz, respectively. ^1^H continuous wave (CW) and two-pulse phase –modulated (TPPM) decoupling schemes of 70 kHz were applied during the π-pulse chains and signal acquisition, respectively. The recycle delay was 2 s and the CP contact time was 1.5 ms.

## 3. Results and Discussions

### 3.1. Secondary Structure Analysis

In our previous work [29], we showed that Vpr solubilized in DPC detergent micelles exhibited a significant fraction of α-helix features. Here, we examined the secondary structure content of Vpr incorporated into lipid bilayer environment. As mentioned in the experimental section, Vpr proteoliposomes were produced by dissolving the protein-lipid film with a buffer and then homogenized with an extruder. The protein concentration in the liposome solution was determined using the extinction coefficient at 280 nm as 19,480 M^−1^cm^−1^, calculated using ExPasy server [38]. We found the incorporation yield of Vpr in proteoliposomes made with DOPG lipid was in a range from 30% to 50%. No significant difference in terms of the Vpr incorporation yield was observed in cholesterol containing proteoliposomes. In contrast, the incorporation yield of Vpr in proteoliposomes made with DOPC alone was not detectable. This observation demonstrated the selective membrane incorporation of Vpr, which was consistent with our previous observation.

**Figure 1:**
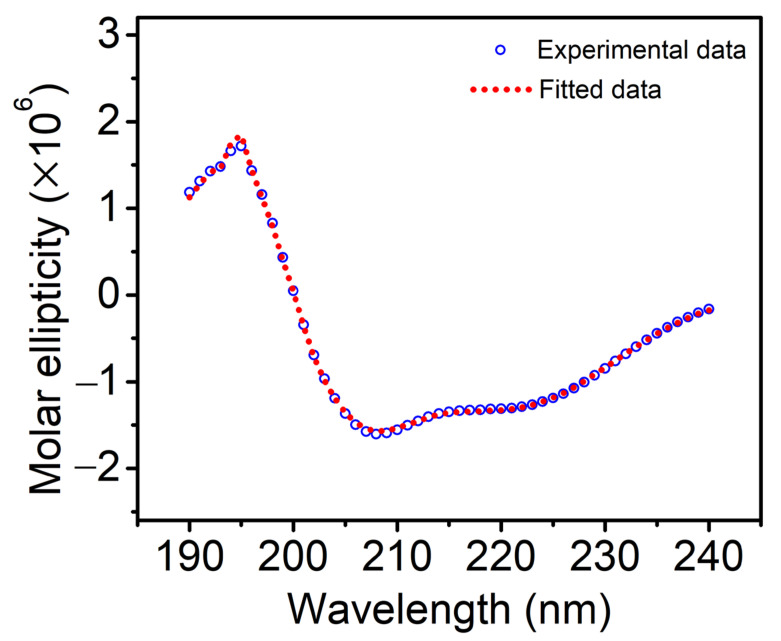
The experimental (open circles) and fitted (dotted line) CD spectra of Vpr incorporated in DOPG proteoliposomes.

**Figure 2:**
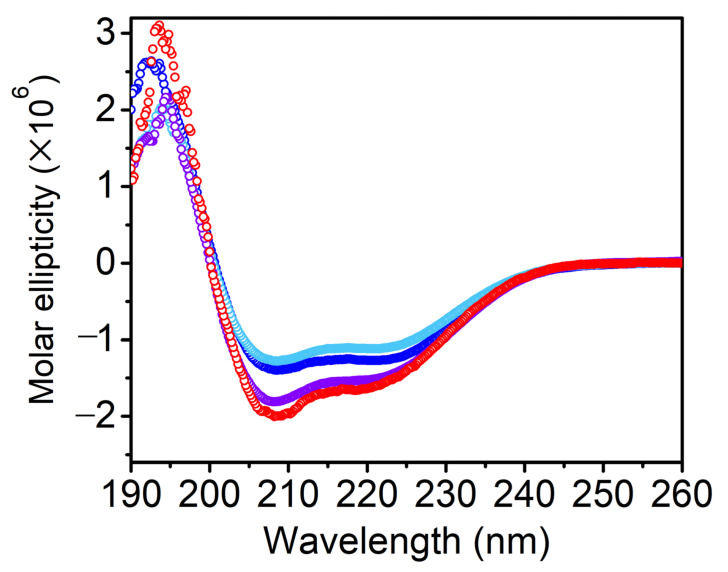
The CD spectra of Vpr solubilized in DPC detergent micelles (red), in DOPG proteoliposomes (purple), and DOPG proteoliposomes in the presence of 5% (blue) and 10% cholesterol (light blue). The ellipticity corresponds to the α-helical content of Vpr is similar in DPC and in DOPG liposome. Vpr maintains similar α-helical structure content in different environments.

The CD spectrum of Vpr incorporated in DOPG proteoliposomes is shown in Figure 1 and the calculated secondary structure content analyzed by DichroWeb using the CDSSTR program and SP175 as a reference set [39] was shown in Table 1. The secondary structure of Vpr incorporated in the DOPG proteoliposomes was found to have similar α-helical structure content as in DPC detergent micelle reported previously [29]. We further included 5% and 10% cholesterol in the DOPG proteoliposomes and examine the secondary structure of the incorporated Vpr. As shown in the CD spectra, Figure 2, we found that similar α-helical feature was still preserved. Therefore, the secondary structure of Vpr in lipid bilayer environment do not change significantly in the presence of cholesterol.

### 3.2. Calcein Release Assay

**Figure 3:**
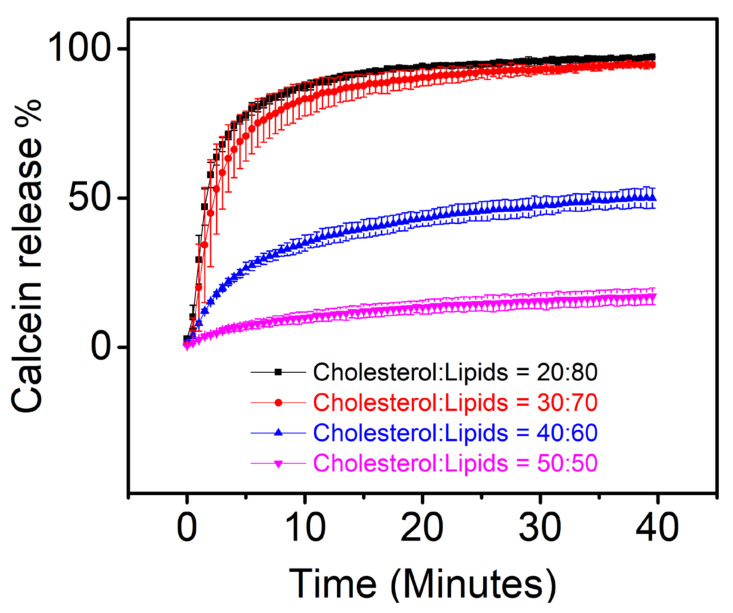
Cholesterol concentration dependent permeabilization of liposomes induced by the membrane binding of His-tagged GB1-fused Vpr. The molar ratio of cholesterol to lipids (DOPC:DOPG = 1:1) in liposomes was increased from 20% to 50%. The reported values are the averages of the triplicate experiments and the standard deviation of each data point is represented as the error bars.

In our previous work, we showed that membrane permeabilization caused by the Vpr binding to membrane was lipid composition dependent. We observed that the calcein release kinetics was increased when the ratio of DOPG in membrane was increased. In the present work, we examined if the concentration of cholesterol in lipid bilayer membrane affects Vpr induced membrane permeabilization. Calcein release assay in Figure 3 reveals that the calcein release kinetics was decreased as the cholesterol concentration in membrane was increased, suggesting that the cholesterol may play a role to modulate the Vpr-membrane interaction. Our result is similar to the previous studies, where the presence of cholesterol in membrane was shown to reduce the membrane permeability induced by a pore-forming peptide [35] and CPPs [36].

### 3.3. NMR Characterization

**Figure 4:**
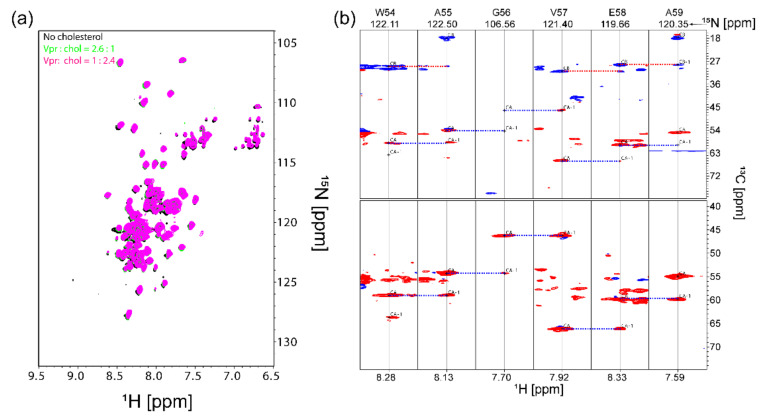
Solution NMR characterizations of [U-^2^H,^13^C,^15^N]-Vpr in [^2^H_25_, 98%] DPC micelles. (**a**) 2D TROSY-HSQC spectra of [U-^2^H,^13^C,^15^N]-Vpr solubilized in [^2^H_25_, 98%] DPC detergent micelles with and without the addition of cholesterol. (**b**) The strips for residues 54–59 from a 3D TROSY-HNCACB (top) and 3D TROSY-HNCA experiments of [U-^2^H, ^13^C,^15^N]-Vpr in [^2^H_25_, 98%] DPC. Red and blue peaks arise from C_α_ and C_β_ nuclei, respectively. The blue and red dashed lines indicate the sequential connection between the strips.

As indicated in calcein release assay, membrane permeability was induced by the binding of Vpr to membrane. The fact that cholesterol plays a role in modulating membrane permeability indicates cholesterol alter the interaction between Vpr and membrane. Here, we attempted to employ solution NMR spectroscopy to examine if cholesterol binds to Vpr specifically. We prepared [U-^2^H,^13^C,^15^N] labeled Vpr protein solubilized in [^2^H_25_, 98%] DPC detergent micelles for solution NMR characterizations. The 2D [^1^H,^15^N] TROSY-HSQC spectrum of Vpr in [^2^H_25_, 98%] DPC micelles in Figure 4a shows that Vpr exhibited significant fractions of α-helix content consistent with the result of CD characterization. It is worth mentioning that the 2D [^1^H,^15^N] TROSY-HSQC spectrum of Vpr stayed unchanged for several weeks, suggesting the sample was of very good stability. The strip plot of TROSY-HNCA and TROSY-HNCACB spectra with the residues assigned from W54 to A59 is shown in Figure 4b to demonstrate the feasibility of protein backbone assignments. By using Talos+ [40], the secondary structure for the segment from W54 to A59 could be predicted based on their chemical shifts. The predictions obtained were well fitted as left-handed α-helical structures in the Ramachandran plot (Figure S3), which is consistent with the structure solved in the protein data bank [41]. No significant chemical shift perturbation was observed when the sample was titrated with cholesterol, as shown in Figure 4a, indicating no evidence of specific cholesterol binding to Vpr solubilized in DPC detergent micelles. The saturation transfer difference (STD) type of experiment [42,43] was not performed due to the overlapping signals of cholesterol and DPC micelles and the low concentration of cholesterol in sample due to the poor solubility of cholesterol.

**Figure 5:**
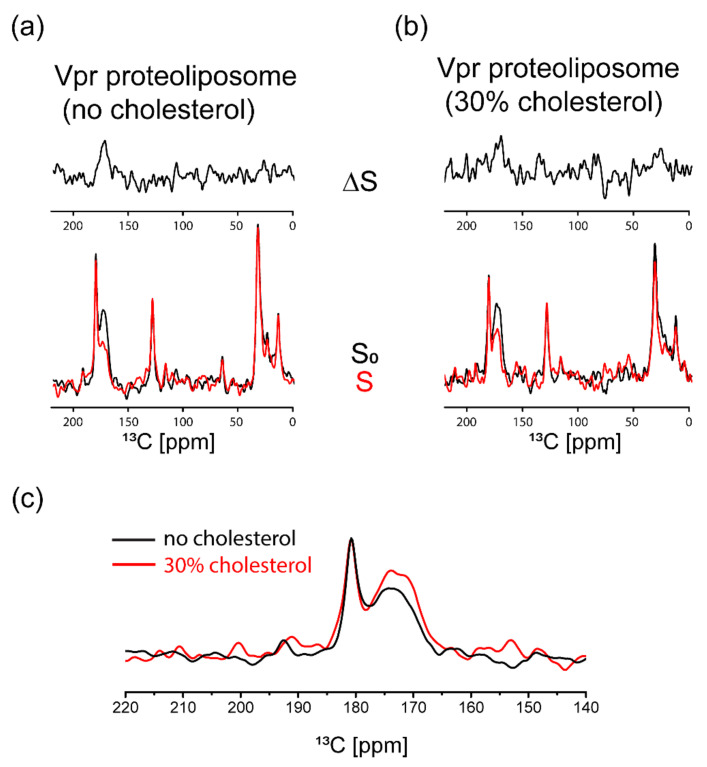
The ^13^C{^31^P} REDOR NMR spectra of [1-^13^C] cysteine selective labeled Vpr embedded in liposomes. (**a**) ^13^C{^31^P} REDOR NMR spectra of [1-^13^C] cysteine selective labeled Vpr embedded in DOPG liposomes. (**b**) ^13^C{^31^P} REDOR NMR spectra of [1-^13^C] cysteine selective labeled Vpr embedded in liposomes (cholesterol: DOPG lipids = 30:70). (**c**) The comparison of the S_0_ spectra of these two samples.

To further examine Vpr in lipid environment, the samples of [1-^13^C] cysteine selective labeled Vpr embedded in liposomes with and without cholesterol were prepared and characterized using ^13^C{^31^P} REDOR NMR spectroscopy. This strategy has been used to study the insertion of HIV fusion peptides in membranes [44,45,46]. Here, we applied REDOR NMR to probe the interaction of Vpr and membrane. A significant ^13^C{^31^P} REDOR dephasing would be observed with 20 ms of the dephasing time if the distance between a ^13^C and ^31^P spin pair is less than 10 Å. In other words, ^13^C{^31^P} REDOR NMR allows us to observe the carbonyl groups of the Cys residue of Vpr that are in proximity of the lipid phosphate groups (<10 Å). The ^13^C chemical shift of carbonyl carbons is around 170 ppm using methylene carbon of adamantane at 38.48 ppm as the external chemical shift reference [47]. It is important to mention that the ^13^C signal observed around 170 ppm is mainly contributed from [1-^13^C] cysteine selective labeled Vpr since no significant contribution of the carbonyl carbons of lipids was observed as shown in Figure S4. The ^13^C signal observed around 170 ppm reflect the chemical environments of the cysteine residue, Cys-76, of Vpr since it is the only one cysteine residue in Vpr sequence (Figure S5). We observed a sharp resonance peak at 181 ppm and a broad resonance peak ranging from 165 to 178 ppm. Typically, the line width of a ^13^C signal from a single-site amino acid residue would be less than 5 ppm [44]. Since different chemical environments will result in different ^13^C chemical shift, the observed broad peak ranging from 165 to 178 ppm indicated the multiple chemical environments which the Vpr Cys-76 residue experiences. Lipid membranes have been shown to play active roles in modulating the functions of membrane associated proteins or membrane proteins [48,49,50,51,52]. Thus, the membrane cannot be viewed as a passive supporting component for the membrane protein due to the couplings between protein and membrane. Rather, we should consider membrane and membrane associated protein as a complex. The broad signal around 170 ppm can be interpreted as the existence of complex Vpr-membrane environments, which may be a result of the existence of multiple Vpr conformations in lipid membrane. While studying the details of the multiple Vpr conformations is interesting, it is out of the scope of the present work. The existence of complex protein-membrane environments has also been observed previously in HIV fusion peptides [44]. As suggested by the REDOR results in Figure 5a,b, some Vpr proteins were in close proximity of the lipid phosphate groups, indicated by the ~40% REDOR dephasing of the broad peak, while some Vpr proteins were not in close contact with the lipids since no dephasing observed for the sharp peak. We further compared the two S_0_ spectra, as shown in Figure 5c. The ratio of the broad peak with respect to the sharp peak increased with the presence of cholesterol in membrane. In addition, the line shape of the broad peak was altered with the addition of cholesterol in the membrane. These results indicated that the distribution of Vpr among these environments was changed in response to the addition of cholesterol in membrane, which may account for our observation of cholesterol modulating the Vpr-membrane interaction as described in Section 3.2.

## 4. Conclusions

Based on the results of calcein release assay, the cholesterol concentration in membrane affects the Vpr induced membrane permeability, suggesting a role of cholesterol in the Vpr-membrane interaction. No obvious evidence of specific cholesterol binding to Vpr was observed in DPC detergent micelles based on the result of the NMR titration experiments. Nonetheless, ssNMR spectroscopy was further used to show that the presence of cholesterol in membrane would affect the distribution of Vpr in the various membrane environments, which may partially account for cholesterol playing a role in the Vpr-membrane interaction. We believe that lipid membrane is as an active component regulating membrane protein functions, rather than just a passive component to support membrane protein. Cholesterol, being an active player in the membrane, can affect the function of membrane protein directly or indirectly.

## Figures and Tables

**Table 1:** Calculated secondary structure fractions of Vpr incorporated in DOPG proteoliposomes.

Helix 1	Helix 2	Strand 1	Strand 2	Turns	Unordered
0.31	0.20	0.03	0.04	0.11	0.32

The secondary structure content was analyzed by DichroWeb using the CDSSTR program and SP175 as a reference set which was optimized for 190–240 nm.

## Data Availability

Not applicable.

## Supplementary Materials

The following are available online at https://www.mdpi.com/article/10.3390/membranes11100784/s1, Figure S1. Examples of the characterizations of Vpr proteins. Figure S2. Intensity distribution of liposomes measured by dynamic light scattering. Figure S3 Ramachandran plot of the assigned protein segments from A55 to E58 shows well defined left-handed α-helical structure predicted by Talos^+^. Figure S4. The ^13^C CPMAS spectrum and ^13^C{^31^P}REDOR S_0_ spectrum of DOPG liposomes. Figure S5. The sequence of Vpr protein and its structure (PDB: 1M8L) with 3 α-helical segments (green) and Cys-76 (magenta) specified.
