# Supplementary material for: Cholesterol Modulates the Interaction between HIV-1 Viral Protein R and Membrane
Source: Membranes (Basel). 2021 Oct 13;11(10):784. doi: 10.3390/membranes11100784 (PMC8539443; doi:10.3390/membranes11100784)
Supplement: Supplementary file 1 [file membranes-11-00784-s001.zip › membranes-1389034-supplementary.pdf]

membranes

Supplementary Materials

Cholesterol Modulates the Interaction Between HIV-1 Viral protein R and Membrane

Chun-Hao Liu [1,2,3], Shing-Jong Huang [4,*] and Tsyr-Yan Yu [3,5,*]

1 Chemical Biology and Molecular Biophysics, Taiwan International Graduate Program, Academia Sinica, Taipei 11529, Taiwan; gerard1057@gmail.com
2 Institute of Bioinformatics and Structural Biology, National Tsing Hua University, Hsin Chu 300044, Taiwan
3 Institute of Atomic and Molecular Sciences, Academia Sinica, Taipei 10617, Taiwan
4 Instrumentation Center, National Taiwan University, Taipei 10617, Taiwan
5 International Graduate Program of Molecular Science and Technology, National Taiwan University, Taipei 10617, Taiwan
* Correspondence: shingjonghuang@ntu.edu.tw (S.-J.H.); tyyu@pub.iams.sinica.edu.tw (T.-Y.Y.); Tel.: +886-2-3366-1152 (S.-J.H.); +886-2-2362-8210 (T.Y.Y.)

Citation: Liu, C.-H.; Huang, S.-J.; Yu, T.-Y. Cholesterol Modulates the Interaction between HIV-1 Viral Protein R and Membrane. *Membranes* **2021**, *11*, 784. https://doi.org/10.3390/11100784

Academic Editors: Akira Naito and Izuru Kawamura

Received: 4 September 2021
Accepted: 10 October 2021
Published: 13 October 2021

Publisher's Note: MDPI stays neutral with regard to jurisdictional claims in published maps and institutional affiliations.

Copyright: © 2021 by the authors. Licensee MDPI, Basel, Switzerland. This article is an open access article distributed under the terms and conditions of the Creative Commons Attribution (CC BY) license (https://creativecommons.org/licenses/by/4.0/).

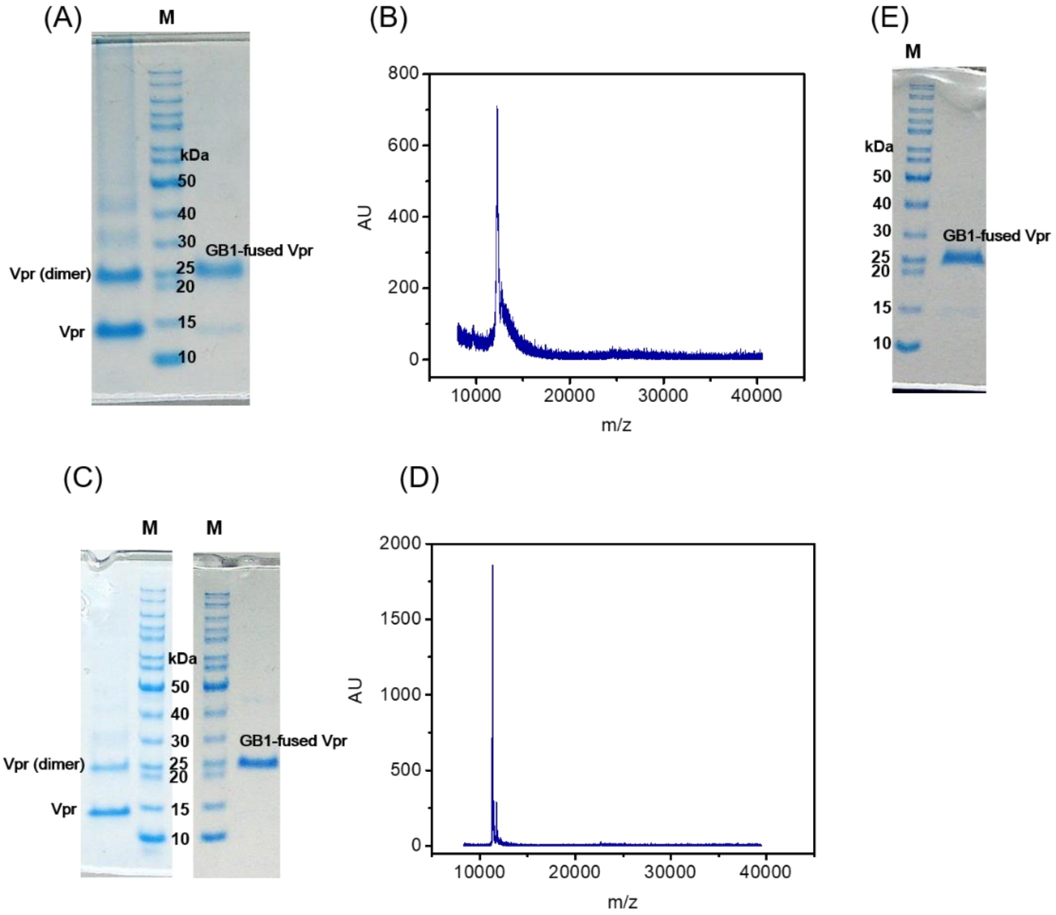

Figure S1. Examples of the characterizations of Vpr proteins. (**A**) The SDS-PAGE characterization of [U-^{2}H,^{13}C,^{15}N] labeled His-tagged GB1-fused Vpr and urea solubilized [U-^{2}H,^{13}C,^{15}N] labeled Vpr. (**B**) MALDI-TOF mass spectrum of [U-^{2}H,^{13}C,^{15}N] labeled Vpr. (observed: 12.37 kDa; theoretical: 12.67 kDa) (**C**) The SDS-PAGE of [1-^{13}C, 99%] Cysteine selective labeled His-tagged GB1-fused Vpr and urea solubilized [1-^{13}C, 99%] Cysteine selective labeled Vpr. (**D**) MALDI-TOF mass spectrum of [1-^{13}C, 99%] Cysteine selective labeled Vpr. (observed: 11.33 kDa; theoretical: 11.33 kDa) (**E**) The SDS-PAGE characterization of natural abundance His-tagged GB1-fused Vpr used for calcein release assay.

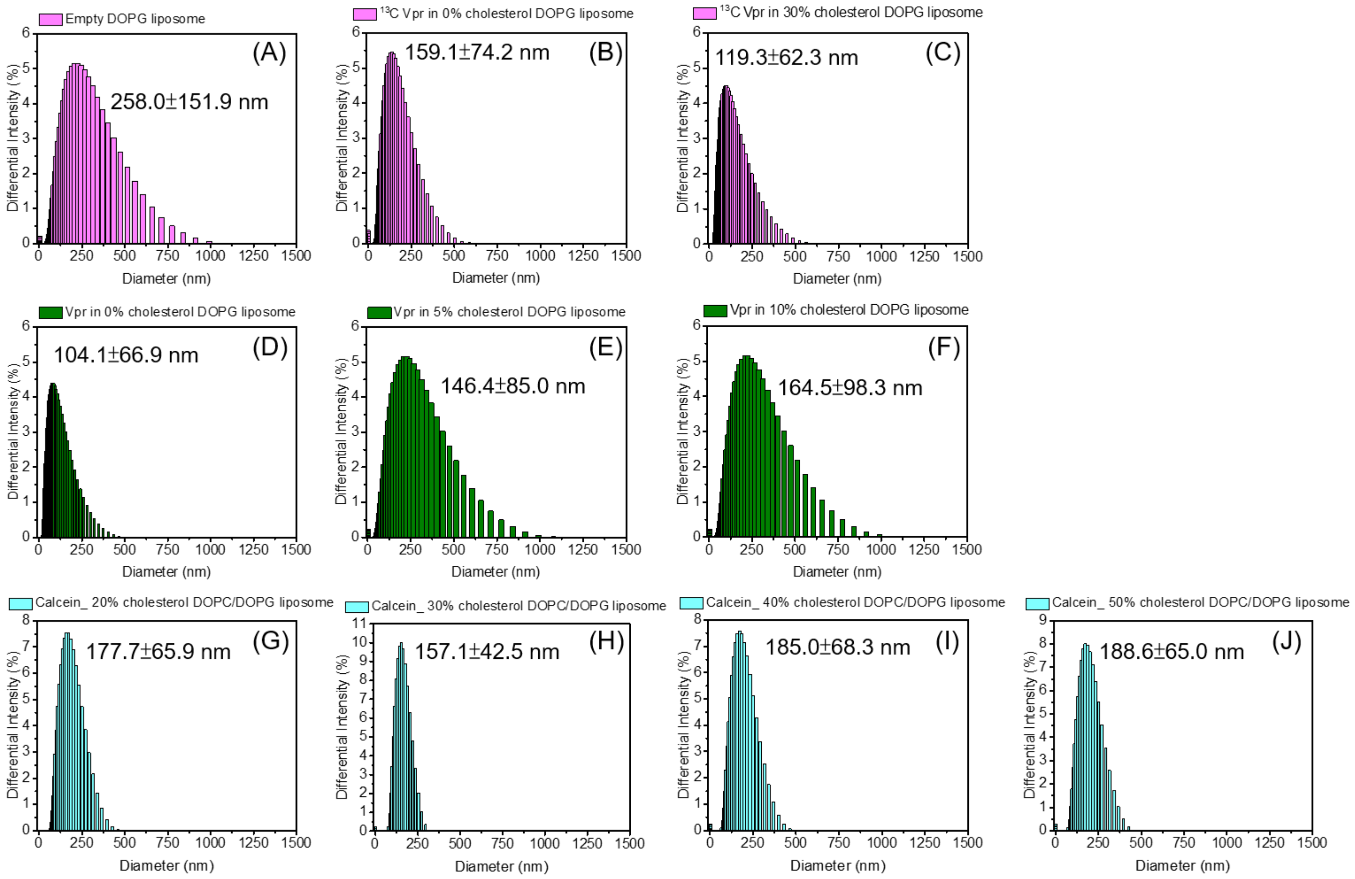

Figure S2. Intensity distribution of liposomes measured by dynamic light scattering. (**A**–**C**) Liposomes used in the ssNMR experiments. (**D**–**F**) Liposomes used in the CD spectra measurements. (**G**–**J**) Liposomes used in the calcein release assay.

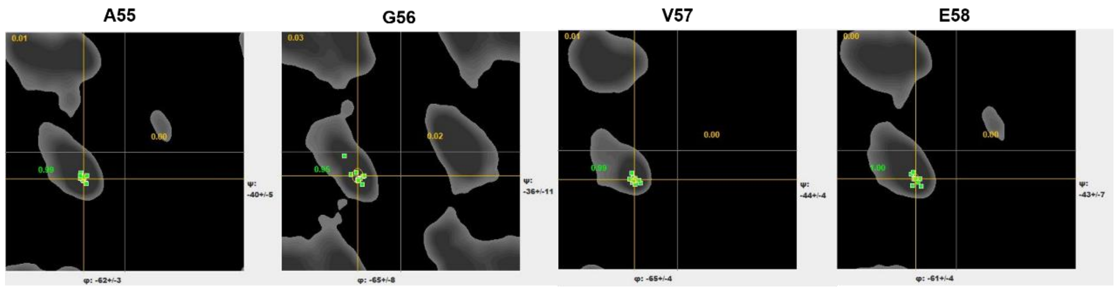

Figure S3. Ramachandran plot of the assigned protein segments from A55 to E58 shows well defined left-handed α-helical structure predicted by Talos+.

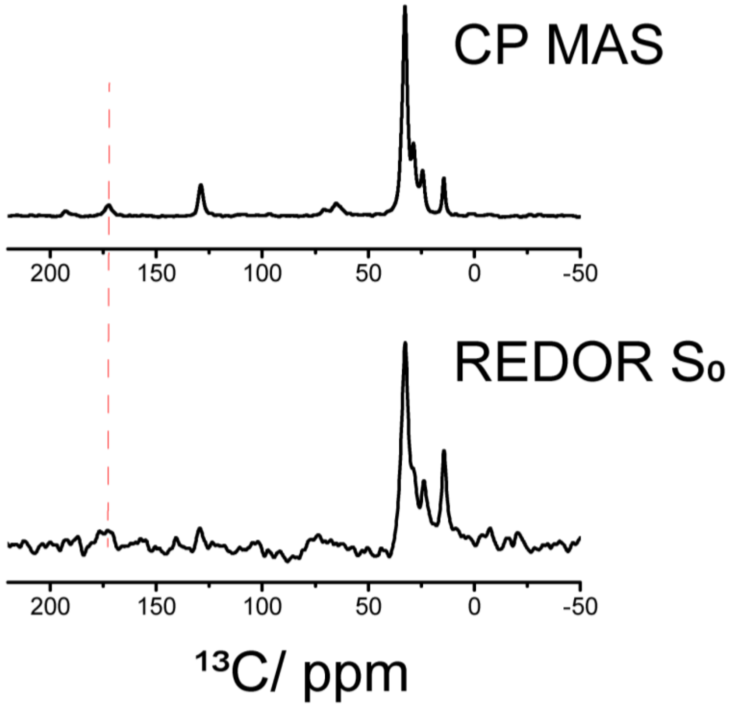

Figure S4. The ^{13}C CPMAS spectrum and $^{13}C\{^{31}P\}$REDOR S_0 spectrum of the DOPG liposome sample. The $^{13}C\{^{31}P\}$REDOR S_0 spectrum was collected with 20 ms evolution time, but without the dephasing pulses turned on, which was the same experimental condition as in Figure 5. It is obvious that the contribution of DOPG lipid to the resonance signal around 170 ppm is trivial.

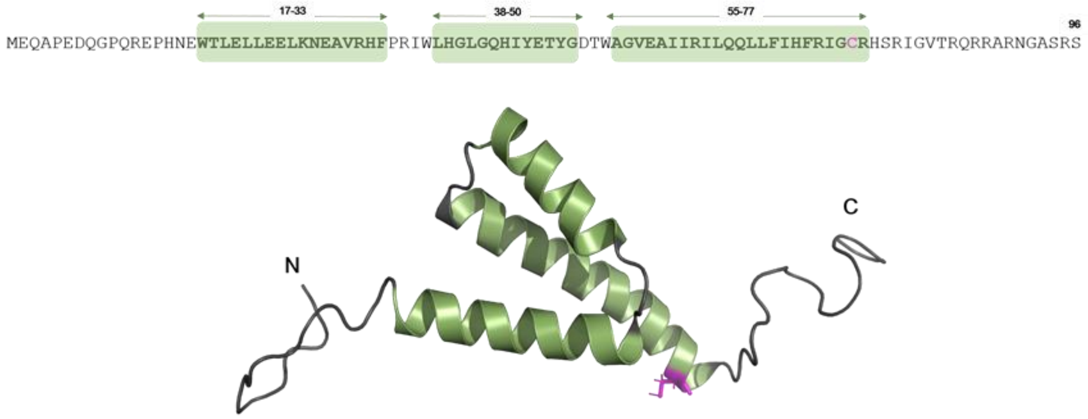

Figure S5. The sequence of Vpr protein and its structure (PDB: 1M8L) with 3 α-helical segments (green) and Cys-76 (magenta) specified.
